# Osteoporosis among Fallers without Concomitant Fracture Identified in an Emergency Department: Frequencies and Risk Factors

**DOI:** 10.4061/2011/468717

**Published:** 2010-11-08

**Authors:** Bente Glintborg, Ulrik Hesse, Thomas Houe, Jensen Claus Munk, Jan Pødenphant, Bo Zerahn

**Affiliations:** ^1^Department of Clinical Physiology and Nuclear Medicine, Herlev Hospital, Herlev Ringvej 75, 2730 Herlev, Copenhagen, Denmark; ^2^Department of Rheumatology and Internal Medicine, 2900 Hellerup, Gentofte Hospital, Denmark; ^3^Danish Medicines Agency, 2300 Copenhagen, Denmark; ^4^Department of Orthopaedic Surgery, 2730 Herlev Hospital, Denmark

## Abstract

We aimed to determine whether the Emergency Department (ED) is a suitable entrance point for osteoporosis screening among fallers without concomitant fracture compared to referral from general practice. Furthermore, to identify factors associated with osteoporosis among fallers. *Methods*. Patients aged 50–80 years sustaining a low-energy fall without fracture were identified from an ED (*n* = 199). Patients answered a questionnaire on risk factors and underwent osteodensitometry. Data was compared to a group of patients routinely referred to osteodensitometry from general practice (*n* = 201). *Results*. Among the 199 included fallers, 41 (21%) had osteoporosis. Among these, 35 (85%) reported either previous fracture or reduced body height (>3 cm). These two risk factors were more frequent among fallers with osteoporosis compared to fallers with normal bone mineral density or osteopenia (previous fracture *P* = .044, height reduction *P* = .0016). The osteoporosis frequency among fallers from ED did not differ from a similarly aged patient-group referred from general practice (*P* = .34). *Conclusion*. Osteodensitometry should be considered among fallers without fracture presenting in the ED, especially if the patient has a prior fracture or declined body height. Since fallers generally have higher fracture risk, the ED might serve as an additional entrance to osteodensitometry compared to referral from primary care.

## 1. Introduction

Osteoporosis is a frequent but widely underdiagnosed condition [1–4]. The frequency of osteoporosis is higher among women and increases steadily with age. Among perimenopausal women, the frequency of osteoporosis is estimated to be 7%–15% [1, 5–7], whereas 30%–40% of all postmenopausal women and 18%–20% of men aged >50 years have osteoporosis [1, 8, 9]. Falling increases the risk of fracture in the osteoporotic individual [10, 11]. The risk of falling increases with age and is more frequent among women than men [12]. One third of all individuals aged 65+ years are susceptible to at least one annual falling episode, and among the 80+ year-old individuals the risk of falling increases to 50% [13]. Previous falls are a strong predictor of new falls [14], and the frequency of fractures associated with falls among the elderly is 6% of which one in six will be a hip fracture [15]. 

The diagnosis of osteoporosis is based on measurement of bone mineral density (BMD) and is defined as a T-score less than −2.5 standard deviations (SD) below the mean value for young healthy individuals [7]. Dual-energy X-ray absorptiometry (DXA) is the most widely used method for bone densitometry. However, this method is costly and not uniformly available worldwide [7]. Referral to DXA is widely based on an individual evaluation of risk factors predisposing to osteoporosis [16–18]. In Denmark, patients are mainly referred for DXA from general practice, and according to previous reports, 21% of referred women have osteoporosis and 34% have osteopenia [19]. Compared to the prevalence of osteoporosis in the general population, the general practitioners appear to have an acceptable ability to select patients at higher risk of osteoporosis [19]. However, fallers frequently present in the emergency department (ED) and in Denmark, 40.000 65+-year old subjects are annually treated in the ED after a fall [13]. Therefore, the ED might represent a feasible alternative or supplement to the osteoporosis screening initiated through primary care, by identifying fallers without fractures. Furthermore, fallers—as noted above—regardless of having no actual fracture still are at higher risk of future fractures. 

Our primary aim was (a) to describe demographics and determine the frequency of osteoporosis among 50–80 years old subjects presenting in an ED after a fall without having sustained a bone fracture and (b) to compare the frequency of osteoporosis among these fallers with that of a group of patients with similar age referred from general practice. A secondary aim was to identify the factors associated with osteoporosis in the group of fallers.

## 2. Patients and Methods

The study was performed at a Danish university hospital (Herlev Hospital) from January 2004 to December 2005. The Local Ethics Committee accepted the study protocol (KA 03097, November 2003), and the study was performed in agreement with the Helsinki declaration. In 2005, the ED at Herlev Hospital had a local catchment area of approximately 200000 citizens and treated 100 surgical or medical patients daily. On average, 60% of treated patients were 50+ years old, and one third of contacts involved fractures, sprains, bruises, lacerations, and so forth. 

Patients were eligible for inclusion in the study if they were aged 50–80 years and if they had attended the ED due to trauma sustained during a low-energy fall. A fall was defined as a sudden, unintentional change in position causing an individual to land at a lower level, on an object or the ground, rather than as a consequence of sudden onset of paralysis, epileptic seizure, or overwhelming external force [20]. We considered a fall as low energy if the maximum displacement was to ground level from a standing position; thus, we excluded falls downstairs, falls from heights, and so forth. Patients were excluded from the study if they had acquired a bone fracture during the actual fall, if they became admitted to hospital after the trauma, or if they already received medical treatment for osteoporosis. Only community-dwelling patients able to communicate in Danish or English were included. Patients with dementia or with diseases making them unable to manage transportation to the hospital were excluded. We aimed to include a predefined number of 200 patients. The inclusion of patients was made on randomly selected days equally distributed over a year in order to facilitate sufficient time and capacity for scanning and to avoid seasonal changes of BMD.

ED files from randomly selected dates were used for prospective inclusion of patients. All patients were contacted by phone by one of the study investigators within 1–3 months after the visit to the ED if they had been treated for lacerations, sprains, or other trauma secondary to a fall. The phone interview was performed using a semistructured interview technique. The patients were questioned in order to secure that they fulfilled the inclusion criteria and that no exclusion criteria were present. Eligible patients received a letter with written information about the study. All participants gave written and verbal informed consent before inclusion. 

All included patients filled in a questionnaire concerning body height reduction since their mid-twenties, lifestyle (exercising habits, dietary habits, and vitamin intake), smoking habits, medication use (patient self-report), alcohol abuse (more than 14 drinks a week for women and more than 21 drinks a week for men (one drink is approximately 12 grams of alcohol)) [21], predisposition to osteoporosis, history of bone fractures within the previous 20 years (proximal humerus, distal antebrachium, femoral neck, thoracic and lumbar spine, or pubic bone), vision, chronic disease, mobility, and employment status. Women additionally answered questions concerning menopausal status and previous childbirths. One of two of the study investigators (BG or BZ) went over the questionnaire with all participants in order to clarify uncertainties and potential misunderstandings.

All patients had their body weight and body height measured by a trained nurse prior to densitometry. DXA scans (Lunar Prodigy, GE Medical Systems, Madison, WI, USA) were performed on both femoral necks and the lumbar spine (L2–L4) in all included patients. The data presented in the following paper are based on computerized numerical BMD values of the femoral neck and the lumbar spine for each patient. A specialist in densitometry evaluated all scans. The T- and Z-scores were defined according to the World Health Organization's recommendations [7]. A T-score below −2.5 SD was considered diagnostic of osteoporosis, and a T-score below −1 SD but higher than or equal to −2.5 SD was diagnostic of osteopenia.

Patients referred from general practice represent by far the largest group among those who are DXA scanned at our department, and the frequency of osteoporosis in this group is thus a suitable reference point. Therefore, we selected 201 subjects routinely referred to the department of clinical physiology, Herlev Hospital from local general practices on the suspicion of osteoporosis. The patients were selected at random in a retrospective manner after the recruitment of fallers was completed. The patients were selected by a study staff member with no knowledge of the purpose of the study. Inclusion criteria were referral during the time period January 2004 till December 2005, age between 50 and 80 years, patients able to self-transport to hospital, and no previous DXA scan. Selection was made as a simple random sample so that all patients in the time period had equal probability of selection. Referral diagnoses, age, height, weight, and T-scores were recorded. As patients routinely referred from general practice do not answer questionnaires or report previous fractures or falls, these data were not available.

## 3. Statistics

Demographic data are reported using descriptive statistics and ANOVA with Bonferroni correction for comparison between normal, osteopenia, and osteoporosis for each gender. Chi-square tests (categorical data) and independent-sample *t*-tests (continuous data) were used for comparison of independent groups of data except when comparing T-scores for men in various age groups between fallers and patients referred from general practice, where a Mann-Whitney U test was used due to low numbers in one of the groups. 

We used stepwise backward logistic regression for analysis of factors associated with osteoporosis among fallers. Age and questionnaire data was transformed into the following categorical variables: age 50–59/60–69/70+ years; perceived body height reduction >3 cm yes/no; family predisposition for osteoporosis yes/no; sedentary work yes/no; regular physical exercise yes/no; previous fracture yes/no; smoking status current/previous/never smoker; able to rise from a chair single handedly yes/no; alcohol abuse (women >14 and men >21 drinks per week) yes/no; body mass index ≤20/>20 kg/m^2^. BMI was also tested as a continuous variable. The risks associated with osteoporosis were described by relative risks (RRs) as RRs are easier to interpret than odds ratios. RRs were estimated from a log-binomial regression analysis only containing factors proven statistically significant in the logistic regression model [22]. 

The ability of associated factors to correctly distinguish fallers with osteoporosis from patients with normal BMD was reported as sensitivity, specificity, positive predictive values (PPVs), and negative predictive values (NPVs). 95% confidence intervals (CIs) were calculated from a score model for binomial confidence intervals [23]. 

Level of statistical significance was *P* < .05. The statistics were calculated with SPSS (version 17.0) and SAS (version 9.0).

## 4. Results

### 4.1. Fallers Identified in the ED

**Figure 1:**
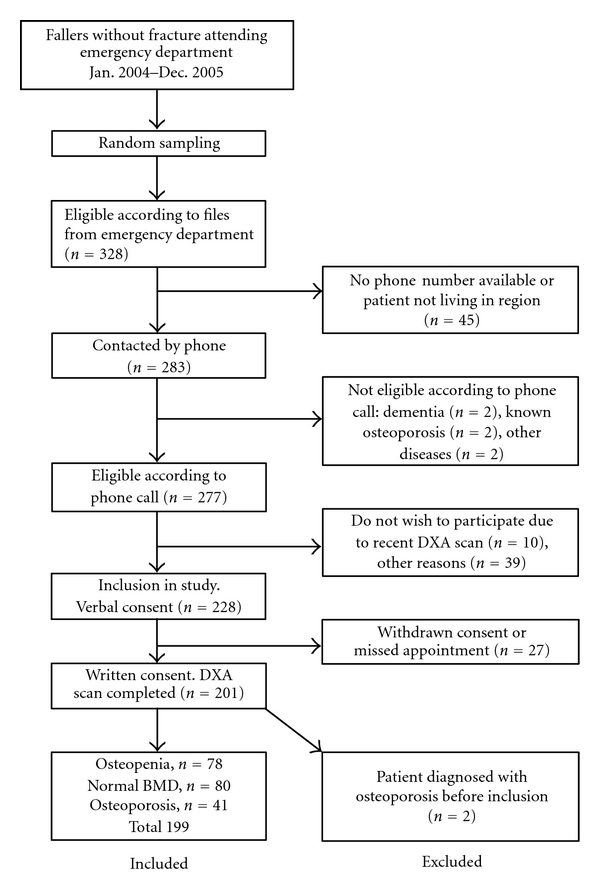
Trial profile: Number of fallers included or excluded in the study.

Patient flow is outlined in Figure 1; 201 fallers (128 women and 73 men) completed the study, underwent a DXA scan, and answered the questionnaire. However, 2 apparently eligible patients revealed having known osteoporosis only after the DXA scan had been completed. Data from these patients were therefore excluded from analysis leaving 199 patients completing the study protocol.

These 199 patients had a median age of 61 years (range: 50–79 years), and 126 (63%) were women. A total of 41 patients (26 women and 15 men) (21%) had osteoporosis, and 78 patients (49 women and 29 men) (39%) had osteopenia. Female fallers with normal T-score were significantly younger, taller, heavier, and had higher BMI than women with osteoporosis. A similar pattern was seen between normal women and women with osteopenia, whereas there was no difference between women with osteoporosis and osteopenia (Table 1). Men with normal T-scores were heavier and had a borderline higher BMI than their osteoporotic counterparts (Table 1).

### 4.2. Fallers Compared to Reference Group

Data from fallers were subsequently compared to data from the group referred from general practice. Age distribution was uniform for both genders when comparing fallers to patients referred from general practice. Fallers had higher body weight than patients referred from general practice. Male fallers had significantly higher BMI than those from general practice, and a similar trend was found for women. Female fallers were significantly taller than those referred from general practice, whereas there was no difference among men (Table 2).

There was an uneven gender distribution between patients referred from general practice and fallers. In total, 73 fallers (37%) were men compared to 27 (13%) in the general practice group (*P* < .001). There was no significant difference in the frequency of osteoporosis among the two groups as a whole, since 41 fallers (21%) had osteoporosis compared to 47 in the general practice group (23%) (*P* = .34). However, the frequency of osteoporosis was higher among men referred from general practice (10 out of 27, 37%) compared to male fallers (15 out of 73, 21%) (*P* < .001). 

**Figure 2:**
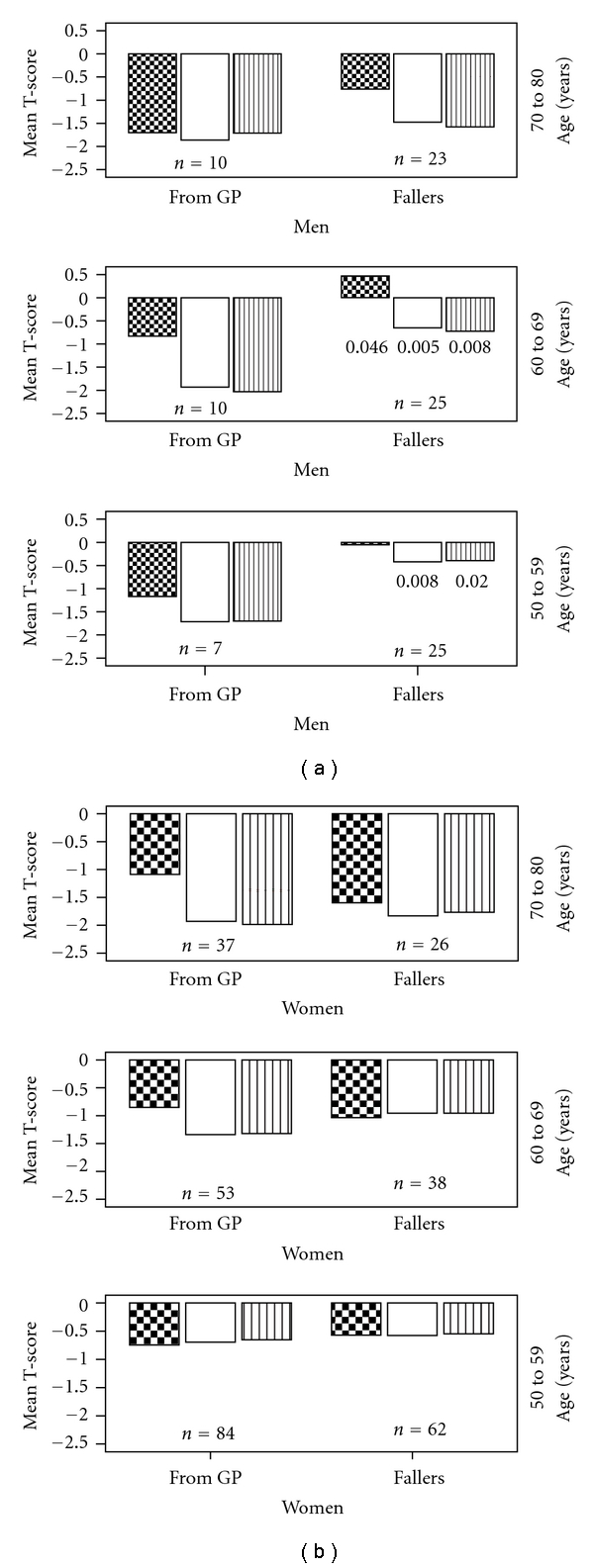
Mean T-score measured among fallers (*n* = 199) compared to patients referred from general practice (*n* = 201) by patient age. Data on men and women are reported separately. Significant differences are marked with *P*-values. Chequered bars: lumbar spine; empty bars: left femoral neck; bars with vertical stripes: right femoral neck.

Comparisons of mean T-scores in different age groups among male and female fallers compared to patients referred from general practice are illustrated in Figure 2. The T-scores of both femoral necks and lumbar spine were significantly higher among male fallers aged 60 to 69 compared to those referred from general practice (T-score fallers: right hip = −0.70 (±1.42); left hip = −0.64 (±1.23), and lumbar spine = 0.47 (±1.93) versus general practice patients: right hip = −2.03 (±0.82), *P* = .008; left hip = −1.93 (±0.85), *P* = .005, and lumbar spine = −0.83 ((±1.02), *P* = .046), whereas the T-score measured in both hips was significantly higher among male fallers aged 60–69 years compared to those referred from general practice (T-score fallers: right hip = −0.40 (±1.51) and left hip = −0.42 (±1.22) versus general practice patients: right hip = −1,70 (±0.71), *P* = .02 and left hip = −1.71 (±0.80), *P* = .008) (Mann-Whitney U test). No significant differences were present among women fallers compared to women referred from general practice in any age group.

#### 4.2.1. Factors Associated with Osteoporosis among Fallers

Questionnaire and anthropometric data from patients with osteoporosis were compared to data from patients with normal BMD/osteopenia in order to identify the factors associated with osteoporosis. According to the multiple logistic regression analysis, the prevalence of osteoporosis was greater among those with one or more previous fractures compared to those with no fracture history (RR 1.9 (95% CI 1.1 to 3.3), *P* = .04), with reduced body height of at least 3 cm compared to those with lesser or no height reduction (RR 2.8 (1.4 to 5.6), *P* < .01), and with increasing patient age (decennium 60–69 (RR 1.9 (0.9 to 4.2)) and 70–80 years old patients (RR 2.7 (1.3 to 5.6)) compared to decennium 50–59, *P* = .04). The remaining variables were insignificant: family predisposition for osteoporosis, sedentary work, regular physical exercise, smoking status, able to rise from a chair single handedly, alcohol abuse, and BMI regardless of dichotomising BMI or not.

A stepwise backward logistic regression analysis was also performed among women (*n* = 126). This analysis added categorical data for parity, previous abortions, early menopause, use of sex hormones, use of hormonal contraception, and uterus surgery as additional factors. Again, the prevalence of osteoporosis was greater among women with reduced body height compared to women with no reduction in height (RR 3.3 (95% CI 1.3 to 8.2), *P* = .02), whereas previous fracture only was a significant factor among the 60–69-year-old ones. The remaining factors were without statistical significance.

Among the 41 patients with osteoporosis, 32 (78%) reported a perceived body height reduction >3 cm since youth, and 13 (32%) reported a previous fracture. Thirty-two (32%) among the 99 fallers reporting a reduction of body height had osteoporosis.  Table 3 shows the ability of self-reported body height reduction and former fracture to correctly distinguish osteoporotic patients from patients with normal BMD/osteopenia [24]. Negative predictive values (NPVs) ranged from 82% to 93%. Six of the patients without risk factors had osteoporosis. Positive predictive values (PPVs) ranging from 30% to 36% indicate that many patients despite the presence of one or more risk factors still had normal BMD/osteopenia.

## 5. Discussion

In this study, 21% of women and of men had unidentified osteoporosis if they were aged 50–80 years and presented in an ED after a fall without current bone fracture. Thus, the prevalence of osteoporosis among ED patients was not significantly different from that of patients referred from primary care in our catchment area. It therefore appears to be as efficient to screen nonfractured fallers presenting in the ED as compared to a population of patients referred from general practice. Moreover, the osteoporotic patients identified from the ED were more frequently normal weight men compared to mainly low-weight women referred from general practitioners. Relatively few men were referred for BMD measurements from general practice (13% versus 37% in the group of fallers), but there was a higher percentage of men with osteoporosis among those referred from general practitioners (37%) compared to the male fallers included from the ED (21%). Thus, screening patients from the ED apparently facilitated the identification of osteoporosis among a group of patients which had a different gender distribution, body height, and body weight compared to patients referred from general practitioners.

Increased screening and treatment of osteoporosis appears to be able to reduce fracture rates [25]. Several guidelines on when to screen for osteoporosis are available [16, 26]. Assessment of osteoporosis should always be considered in patients having experienced a fragility fracture [27–30]. The US Preventive Services Task Force recommends routine DXA screening among 65+-year-old women and among younger women having risk factors [31, 32], whereas the use of DXA among men and perimenopausal women is still a matter of debate [33]. However, more restricted screening strategies are often applied in countries with fewer health care resources and limited access to osteodensitometry [17, 34]. Several fallers in our study had previous fractures potentially related to osteoporosis without having had subsequent bone densitometry. Thus, the implementation of guidelines in clinical practice is not yet sufficient in our particular catchment area (Capital Region of Denmark). This may be due to the frequent involvement of several different branches of health care professionals and lack of consensus on who is primarily responsible for the diagnosis of osteoporosis [29]. More attention on this subject is necessary both among physicians in primary care and indeed also among the ED physicians or orthopaedic surgeons initially managing the fracture [29, 35–37]. 

According to a previous study performed in our department, the general practitioners have an acceptable ability to identify patients at increased risk of osteoporosis compared to the background population [19]. To our knowledge, no studies have previously addressed osteoporosis screening of fallers presenting in the ED. As shown in the present study, two single questions regarding body-height reduction and previous fractures were helpful in order to identify the fallers with an increased risk of osteoporosis: 35 of the 41 patients (85%) with osteoporosis had a previous fracture or a perceived body-height reduction of more than 3 cm since early adulthood. The high negative predictive value of 93% illustrates that the advantage of risk factor assessments mainly is to preclude low-risk patients from further osteoporosis screening [26]. Among the 199 included fallers, 83 had no previous fractures or a reduction in body height. If these 83 subjects were excluded from further screening, 6 osteoporotic patients would be missed, but the osteoporosis prevalence among the remaining 116 fallers would increase (35/116 = 30%). Furthermore, falls are associated with better compliance with bisphosphonate treatment [38], which in turn means that focusing treatment on osteoporotic individuals with falls will improve the efficacy of treatment. It can be argued that establishing falls clinics may remove the need for the ED to serve as an entrance point for osteoporosis screening. However, referral to a falls clinic is most often from general practice [39]. Subsequently the ED may still be an important alterative referral point.

Our study has some limitations to consider. Since the study is cross-sectional and only includes 199 fallers, it does not qualify to describe or capture the entire range of risk factors for osteoporosis among nonfractured fallers. The group of patients referred from general practice was not tested with the full questionnaire, so the frequency of fallers in this group is not known. Ideally, a group of healthy volunteers should serve as a reference group. We chose to use patients referred from general practice instead because this group of patients is a more relevant comparison when evaluating clinical practice. This may also have caused a selection bias. Furthermore, our study was performed in a single ED, and our results may not be generalised to other settings or uptake areas. 

In conclusion, the ED might serve as an entrance point for osteoporosis screening among 50–80-year-old fallers because fallers in general have a higher risk of fracture. Bone densitometry should as a minimum be offered to patients presenting with falls without concomitant fracture if the patient has a prior fracture or a decline in body height. Scanning of all fallers from the ED would identify osteoporotic individuals with the same frequency as patients referred from general practice. As the gender distribution and body height/weight among ED fallers differs from the patients referred from general practice, the ED might serve as an important additional entrance point to bone densitometry and osteoporosis screening including more men and of course fallers.

## Figures and Tables

**Table 1:** Demographic data on fallers included from emergency department, *n* = 199, according to osteoporotic status. Results from ANOVA with Bonferroni correction for comparison between groups.

		Baseline characteristics			Results from ANOVA, *P*-values			
		Normal	Osteopenia	Osteoporosis	Overall *P *	Normal versus osteopenia	Normal versus osteoporosis	Osteopenia versus osteoporosis
Women	Height mean (SD)	167 (7)	164 (6)	160 (7)	<.0001	.06	<.0001	n.s.
	Weight mean (SD)	78 (16)	72 (15)	63 (14)	<.0001	.054	<.0001	n.s.
	BMI mean (SD)	28 (6)	27 (6)	25 (5)	.04	n.s.	.03	n.s.
	Age mean (SD)	59 (7)	63 (9)	66 (8)	<.0001	.04	<.0001	n.s.
	N (%)	51 (41)	49 (39)	26 (21)	—	—	—	—
Men	Height mean (SD)	177 (7)	177 (7)	175 (7)	n.s.	n.s.	n.s.	n.s.
	Weight mean (SD)	90 (17)	85 (13)	77 (12)	.02	n.s.	.02	n.s.
	BMI mean (SD)	29 (5)	27 (4)	25 (4)	.050	n.s.	.049	n.s.
	Age mean (SD)	63 (9)	64 (9)	68 (8)	n.s.	n.s.	n.s.	n.s.
	N (%)	29 (40)	29 (40)	15 (21)	—	—	—	—

**Table 2:** Demographic data on fallers included from emergency department (*n* = 199) compared to patients referred from general practice (*n* = 201). Data on women and men are reported separately. Results from independent sample *t*-test.

		Women			Men	
	Fallers *n* = 126 Mean (SD)	General practice *n* = 174 Mean (SD)	Fallers versus general practice *P*-value	Fallers *n* = 73 Mean (SD)	General practice *n* = 27 Mean (SD)	Fallers versus general practice *P*-value
Age, years	62.0 (8.2)	62.7 (8.7)	n.s.	64.6 (8.9)	66.9 (8.9)	n.s.
Weight, kilo	72.6 (16.0)	66.7 (13.9)	.001	85.4 (15.3)	77.5 (11.2)	.006
Height, cm	164.2 (7.3)	161.2 (6.6)	.001	176.3 (7.0)	174.7 (7.5)	n.s.
Body mass index, kg/cm^2^	27.0 (5.8)	25.7 (5.3)	.06	27.4 (4.6)	25.3 (2.8)	.03

**Table 3:** The ability of two selected factors to correctly identify fallers with osteoporosis. Sensitivity, specificity, positive predictive values (PPVs), and negative predictive values (NPVs).

Associated factor	True positive (TP)	True negative (TN)	False positive (FP)	False negative (FN)	Sensitivity	Specificity	PPV (95% CI)	NPV (95% CI)
Body height reduction (self-reported)	32	91	67	9	78	58	32 (28–36)	91 (89–93)
Previous fracture (osteoporotic localisation)	13	135	23	28	32	85	36 (27–45)	82 (64–100)
Self-reported body-height reduction >3 cm and/or fracture	35	77	81	6	85	49	30 (27–34)	93 (92–94)

Sensitivity = TP/(TP + FN): ability to correctly identify osteoporotic fallers.

Specificity = TN/(TN + FP): ability to correctly exclude fallers with normal BMD/osteopenia.

PPV = TP/(TP + FP).

NPV = TN/(TN + FN).
